# Can COVID-19 mark a tipping point for home-based telework? Conflict between untact technology and rigid institutions in Korea

**DOI:** 10.3389/fpubh.2023.1289809

**Published:** 2023-11-29

**Authors:** Joonmo Cho, Sanghee Lee, Byungjin Park

**Affiliations:** ^1^Department of Economics, Sungkyunkwan University, Seoul, Republic of Korea; ^2^Department of Consilience, Korea Polytechnic University, Siheung, Republic of Korea; ^3^Department of Economics, HRD Center, Sungkyunkwan University, Seoul, Republic of Korea

**Keywords:** COVID-19, home-based telework, untact technology, sustainable utilization, rigid institution

## Abstract

**Background:**

Previously, Korea showed a passive attitude toward home-based telework; however, this stance rapidly changed after the COVID-19 pandemic. Sustaining home-based telework entails adjusting productivity conditions, introducing performance-based evaluations, and modifying employment rules, as required by the Korean Labor Standards Act, which demand the consent of most workers. This study aims to explore the societal and institutional shifts necessary for ongoing home-based telework post-pandemic.

**Methods:**

This study discusses the sustainability of home-based work based on survey data and materials from institutions and previous research. It used data from the Workplace Panel Survey provided by the Korea Labor Institution for 3 years (2015, 2017, and 2019) to examine the status of home-based work and business responses. It also addresses legal issues related to changes in working conditions and worker-management agreements resulting from telework implementation. Legal aspects of telework are explained using relevant sections of Korea’s labor laws.

**Results:**

To establish home-based telework as a working method relevant to the Fourth Industrial Revolution after the pandemic, essential discussions are needed regarding its fundamental applicability to specific job sectors. Moreover, to activate home-based telework without deteriorating working conditions, achieving agreement between workers and management is imperative. However, legal complexities necessitate systemic changes for effective resolution. For the sustainable continuity of telework, a blend of societal awareness and institutional transformations is indispensable.

**Discussion:**

The growth of home-based telework through untact technology expansion is hindered by inflexible Korean labor laws, judicial precedents, and worker-management relations. The absence of necessary legal and organizational changes could lead Korea to revert to pre-pandemic norms or slow implementation. Initially prevalent in IT, home-based telework has expanded across sectors due to the pandemic. Leading the “new normal,” companies creatively enhance productivity through telework, but rigid systems and outdated cultures could impede post-pandemic progress.

**Conclusion:**

The study highlights the need for forward-looking institutional changes and adaptation to advancing technology. It provides valuable insights for organizations and policymakers to optimize work dynamics and enhance employee and employer well-being in the post-COVID-19 era.

## 1 Introduction

With the rapid advancement of technology in contemporary society, people can now connect through the internet almost instantaneously. This has generated a growing interest in Telework, which refers to the practice of independently performing tasks at a location and during times not specifically associated with a particular company or institution (1). Telework is a work arrangement that comes with various advantages and disadvantages. One of the main advantages of telework is enhanced productivity, increased job satisfaction, and reductions in commuting time and expenses (2–4). Moreover, it has been shown to offer mental benefits, such as increased freedom in time management, reduced stress, and an improved work-life balance (3, 5).

Conversely, telework has encountered criticism stemming from its departure from the conventional office environment. These concerns encompass social isolation, the absence of face-to-face communication, and a lack of support from mentors and colleagues (2, 6–11). These criticisms also extend to apprehensions about overworking when working from home (12), and the effectiveness of productivity, which may not necessarily improve, depending on the nature of the tasks (13). Furthermore, telework faces limitations regarding its suitability across all industries, given its reliance on factors like the type of occupation, work format, and the voluntariness of participation (14, 15).

In response to COVID-19, companies are making significant efforts to prevent epidemics and preserve employment through various measures, including telecommuting and taking paid and unpaid leave (16–19). Companies have developed home-based telework manuals that incorporate information technology (IT) solutions and security guidelines to facilitate telecommuting (20–22). Concurrently, the government is actively contributing to this endeavor by providing the essential infrastructure required for telecommuting and offering wage subsidies to support companies in their efforts to prevent unemployment and assist the economically inactive population.

More specifically, while large companies in Korea can develop the necessary infrastructure, including the technology for implementing home-based telework on their own, it poses a challenge for small- and medium-sized enterprises (SMEs) to undertake such investments. To address this disparity, the government has implemented policies to support smaller enterprises, albeit with certain limitations. The COVID-19 pandemic has prompted a rapid and substantial increase in home-based telework, which has not been extensively practiced in Korea. Large companies adopted it as a self-rescue measure, and the government introduced it as a support policy for SMEs affected by the pandemic. Consequently, Korea’s IT industry has made significant advancements, with IT solutions such as Zoom and Webex sufficiently upgraded to accommodate the demand for home-based telework. This transformation has led many technical experts to predict that the new technologies of the Fourth Industrial Revolution will drive the flexibilization of work-hour systems, largely influenced by home-based telework. Moreover, the integration of untact technology with well-designed online connection systems has given rise to “ontact” technology, which bears a resemblance to home-based telework practices.

Many questions have arisen regarding the potential expansion of home-based telework as a future working method post-COVID-19 (6, 23–25). Those who predict growth often emphasize its technical aspects and view it as a valuable experimental option, albeit with certain limitations in terms of its duration. However, as the saying goes, “the devil is in the detail,” we must consider whether the necessary technical changes and the changes required in the work environment can occur smoothly or if they might clash and create obstacles. The introduction of home-based telework will bring about changes in the working methods of individual workers or worker groups, which must be addressed within the framework of Korean laws. Thus, a proper procedure for managing these changes is essential. If this procedure requires the consent of individual workers or worker groups, it becomes crucial to assess whether home-based telework can be expanded by effectively overcoming these consent-related challenges. If a rigid institutional barrier hinders expansion, it will be difficult to progress with the adoption of teleworking on a larger scale. Moreover, the core of the consent procedure hinges on whether workers and management can jointly select a cooperative strategy for introducing home-based telework that results in a win-win situation. The successful expansion of telework largely depends on whether Korean worker-management relations can facilitate strategic collaboration between the two parties.

This study aimed to comprehensively examine the impact of COVID-19 on the labor market in Korea and the responses of Korean companies to the pandemic. We conducted a comparative analysis between the pre-pandemic period, characterized by a passive approach toward home-based telework, and the post-pandemic period, which witnessed a rapid increase in home-based telework. Through this comparison, we sought to identify and analyze the effects of various institutional factors that play a role in determining the post-pandemic sustainability of home-based telework. Our investigation also delves into whether any consent-related procedures, mandated by labor laws and regulations, pose obstacles to adopting home-based telework. We will explore the possibility of reaching a mutual agreement between workers and management to facilitate the required consultation process for effectively implementing telework. Based on our findings, we aim to provide insights into a potential scenario in which companies’ competitiveness and workers’ quality of life could be simultaneously enhanced through integrating untact technology with the labor market system in each country post-COVID-19 pandemic. This study seeks to offer valuable information for fostering harmonious and strategic collaborations between stakeholders, leading to positive outcomes for businesses and workers.

## 2 Method

In this study, we utilize survey data from workplace panels and the content of literature on home-based telework to discuss the changing perceptions and circumstances of telework before and after the COVID-19 pandemic. We also examine the challenges associated with telework usage. Additionally, we discuss the necessity of consensus between labor and management on implementing telework and address legal matters related to working conditions. Through this multifaceted approach, this study aims to answer the following questions:

- Can home-based telework, which saw a significant increase in demand during the COVID-19 pandemic, continue to be used as a fundamental employment system even after the pandemic?
- Is there a need for societal and institutional consensus for home-based telework to establish itself as an employment system?
- What are the societal and institutional issues that need to be addressed to promote the activation of home-based telework, and in what direction should we move?

To facilitate this discussion, we have analyzed responses regarding home-based work from the Workplace Panel Survey Data. Furthermore, we have utilized survey results primarily from the Korea Chamber of Commerce and The Ministry of Employment and Labor, along with findings from published papers by these institutions. We have also cited foreign references under the corresponding tables and figures. To elucidate the legal aspects of telework, we have extracted relevant sections from Korea’s labor laws, followed by explanations and discussions to provide a comprehensive understanding.

### 2.1 Data

The data used in this study was collected from the Workplace Panel Survey Data (WPS) obtained from the Korea Labor Institution. WPS is a dataset that conducts biennial surveys on employment, labor demand, human resources, and training in various workplaces. In this research, we used WPS data from 2015, 2017, and 2019 to examine the perception and utilization of home-based telework in businesses before and after the occurrence of the COVID-19 pandemic, stratified by company size and industry classification. The WPS survey is not conducted in the same year but rather the following year. WPS2019 was conducted during the COVID-19 pandemic, and in addition to the regular questions about home-based work, specific questions about how businesses responded to the COVID-19 pandemic were added. In this study, we utilized both the standard questions about home-based work and the pandemic-related questions to conduct our analysis.

The following are the specific questions posed to businesses in the WPS regarding home-based work:

- As of the end of the previous year, did your business operate a telecommuting or remote work system?
- In response to the spread of the COVID-19 virus, did your business utilize flexible work systems (telecommuting, flextime system, remote work, flexible work arrangements system)?
- Which flexible work systems did your business employ?

The responses to the first question are categorized into (1) Yes, employees are actually using it, (2) Yes, but no employees are utilizing it, and (3) No. The second question is answered with a simple Yes or No. In the case of the third question, respondents may choose multiple options, indicating the specific flexible work systems they have implemented, which include telecommuting, flextime system, remote work, flexible work arrangements system.

## 3 Results

### 3.1 WPS results

#### 3.1.1 The operation status of telecommuting and remote work systems

**Figure 1:**
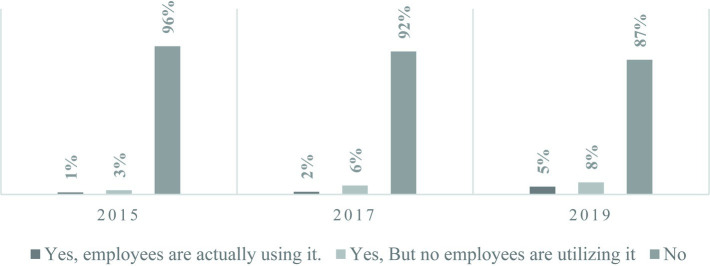
The operation status of telecommuting and remote work systems before the COVID-19 pandemic. The WPS survey is conducted in the year following the reference year, and the responses to the questions mentioned above are based on the status as of the year prior to the survey year.

Figure 1 represents the response results regarding the operation of telecommuting and remote work systems in businesses before the pandemic. In 2015, out of 3,431 businesses surveyed, only about 1% or 50 businesses reported that their employees were actually using telecommuting and remote work systems. In both 2017 and 2019, approximately 2 and 5% of all businesses, respectively, indicated that employees were utilizing these systems. While the number of businesses implementing these systems has gradually increased, the actual usage by employees has not seen a significant rise.

**Figure 2:**
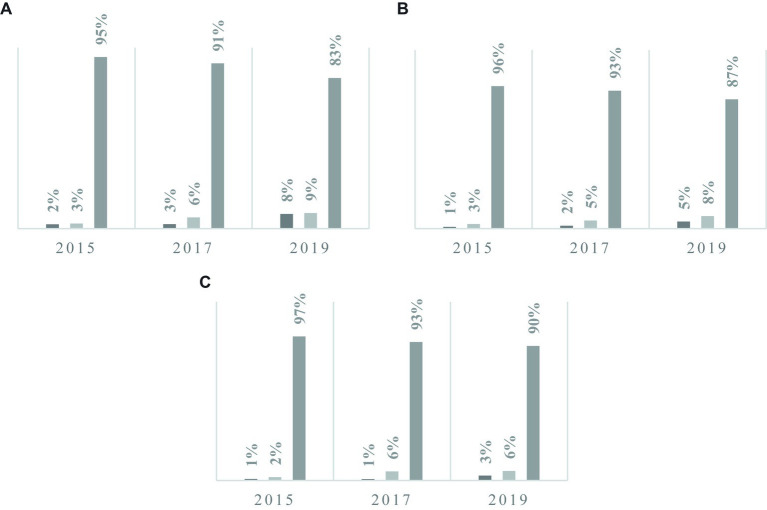
The operation status of telecommuting and remote work systems by business size. **(A)** represents businesses with 300 or more employees, **(B)** represents businesses with 50–299 employees, and **(C)** represents businesses with fewer than 50 employees. The bar graph displays the proportions of responses in the following order from left to right: (1) Yes, employees are actually using it, (2) Yes, but no employees are utilizing it, and (3) No.

Figure 2 displays the operation status of telecommuting and remote work systems according to the size of businesses. It indicates that the actual usage proportion for telecommuting is consistently below 10% across all business sizes. In large-scale enterprises with 300 or more employees, the proportion of remote and telecommuting system implementation gradually increases, reaching up to 17%. However, it is evident that as the size of businesses decreases, the proportion of telecommuting system implementation decreases as well.

**Figure 3:**
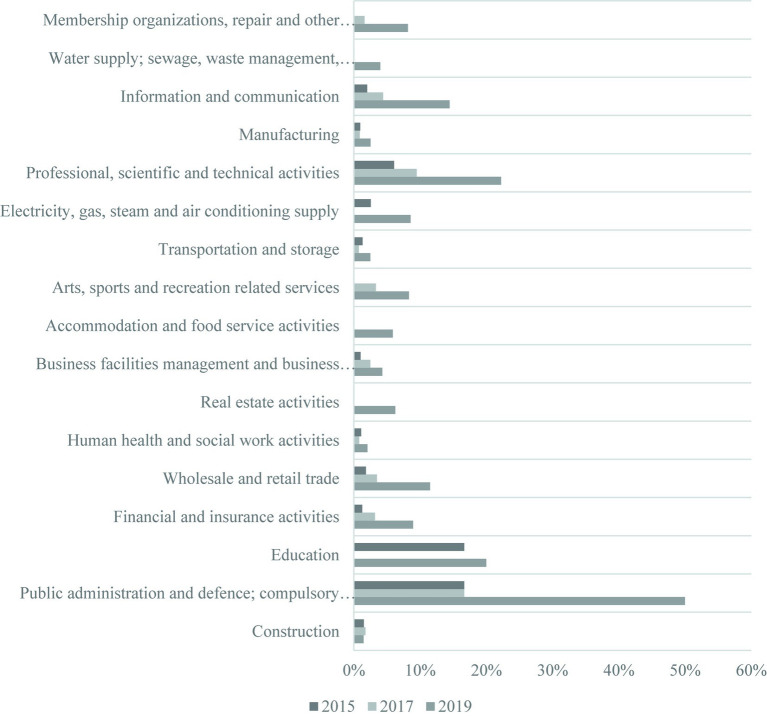
The proportion of businesses, categorized by industry classification, where employees have responded that they are actually utilizing remote and telecommuting work. The above industry classification is based on the 9th Korean Standard Industrial Classification.

Figure 3 illustrates the proportion of businesses, categorized by the Korean Standard Industrial Classification, where employees have responded that they are utilizing remote and telecommuting systems. Notably, the utilization of telecommuting systems is relatively higher in industries such as public administration and defense; compulsory social security, Education, and Professional, scientific, and technical activities. However, the overall introduction of telecommuting systems in various industries appears to be quite low, indicating a concentration of telecommuting system usage in specific sectors.

#### 3.1.2 The implementation status of flexible work systems after the pandemic.

**Figure 4:**
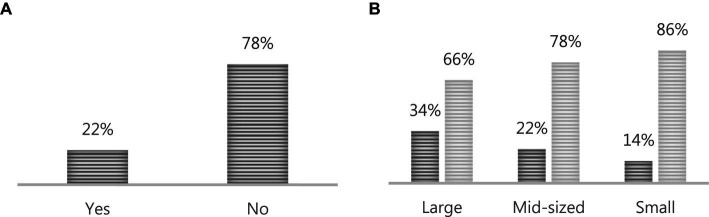
The adoption of flexible work systems for COVID-19 pandemic response. **(A)** Represents the responses from the entire set of businesses, while **(B)** indicates the responses categorized by business size.

To prevent the spread of the coronavirus after the pandemic, businesses aimed to maintain their operations by implementing Flexible Work Arrangement systems, Telecommuting systems, Flextime systems, or Remote Work systems. Figure 4 presents the response results regarding whether businesses adopted flexible work systems in response to the COVID-19 pandemic. Out of 2,795 total businesses, approximately 22%, or 622 businesses, implemented flexible work systems to combat the coronavirus. When analyzed by business size, it is evident that larger enterprises, at around 34%, adopted flexible work systems most extensively for COVID-19 prevention, while smaller businesses exhibited a lower level of response to pandemic control measures.

**Figure 5:**
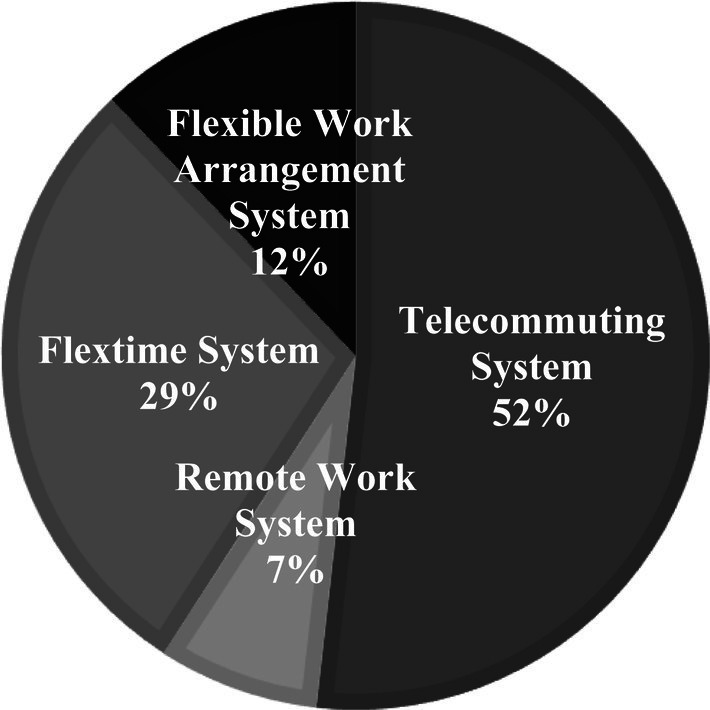
The proportion of flexible work systems implemented for COVID-19 pandemic control.

Figure 5 illustrates the proportion of businesses that have adopted flexible work systems for COVID-19 pandemic control and the distribution of their utilization of various work systems. Telecommuting and remote work systems, at approximately 59%, appear to be the most preferred work systems that businesses have chosen for COVID-19 pandemic prevention.

The actual usage proportion of telecommuting and remote work systems before the COVID-19 pandemic was shown to be quite low, not exceeding 10%. With the increased demand for telecommuting and remote work systems due to the onset of the COVID-19 pandemic, approximately 22% of all businesses introduced flexible work systems, resulting in an increase in usage. However, when viewed holistically, the utilization of telecommuting and remote work systems remains at a relatively low level. It also varies significantly based on business size and industry type.

### 3.2 Companies’ response to COVID-19 and the adoption of home-based telework

In response to the COVID-19 pandemic, many countries, including Korea, implemented and expanded home-based telework as a precautionary measure to mitigate the risk of infection while ensuring that economic activities continued to some extent (19, 22, 26). This approach was particularly suitable for jobs that did not require physical labor at production sites (27). Major companies, such as Amazon and AT&T, responded by instructing their employees to work from home whenever possible (28, 29). In Korea, home-based telework has grown significantly (30). For instance, at the beginning of the new semester, the Korean government allowed students to attend all lessons from home online until the COVID-19 pandemic subsided (31).

#### 3.2.1 Before the pandemic

Korea has actively promoted the implementation of a flexible working system, including home-based telework, to achieve a better work-life balance, addressing the declining birth rate and reducing long working hours. However, despite these efforts, the adoption of flexible working systems, particularly home-based telework, has not expanded as intended. As indicated in Table 1, the utilization of home-based telework remains the lowest among various flexible working methods in Korea, and its introduction rate is considerably lower than that in other countries.

**Table 1:** Introduction rates of a flexible working system.

	Dimensions of flexible working system (%)			
	Korea	Europe	USA	Japan
Home-based telework	3.0	Germany 12 Belgium 20 Sweden 32	38.0	11.5
Flexible working hours	9.2	–	–	52.8
Staggered commuting	12.7	66.0	81.0	–
Part-time	11.3	69.0	36.0	–

Korea Chamber of Commerce (32). This table re-quotes the data compiled by the Korea Chamber of Commerce. “Survey on Introduction of a Flexible Working System by Companies,” Korea Chamber of Commerce. Accessed May 21, 2020. http://www.fki.or.kr/Common/ Download.aspx?id = 2dfd975c-5e1f-48aa-a550-595211ae3d8f. Data provided in this table were extracted from four sources: Korea: Kim et al. (33). Europe: Eurofound, “European company survey,” (2013). USA: Kenneth Matos and Ellen Galinsky, “2014 National Study of Employers,” Society for human resource management, (2014). Japan: The Ministry of Health, Labor, and Welfare, “2014 Comprehensive Survey on Employment Conditions (Flexible Working Time System),” (2014) Decision of Japanese Cabinet Meeting.

“–” indicates that no data are available due to statistical limitations. However, the values shown in the home-based telework column for Europe are the introduction/use rates published by the ILO. The introduction rates of the working method using T/ICTM which enables telecommuting such as home-based telework in other European countries is shown to be 28% in Finland, 12% in France, 15% in the Netherlands [see Messenger et al. (34)]. The Ministry of Employment and Labor. Home-based telework includes telecommuting other than home-based telework.

The promotion of a flexible working system policy in Korea, including recommendations for home-based telework, is ongoing. However, the actual adoption rate of such a system has seen only a marginal increase over the years and has even declined in certain periods. Table 2 provides data on the percentage of different types of flexible working systems used over time, with telecommuting, including home-based telework, displaying a decreasing trend.

**Table 2:** Utilization of flexible work system by type.

	Aug. 2015	Aug. 2016	Aug. 2017	Aug. 2018	Aug. 2019
Home-based telework and telecommuting	7.3	7.3	5.6	4.7	4.3
System of working reduced hours	0.4	7.8		15.1	17.1
Staggered commuting system	42.0	38.1	38.4	33.2	33.7
Selective working hour system	33.2	31.6	31.9	32.3	30.4
Flexible working hours system	26.3	23.7	26.0	27.3	32.0
Other (discretionary work system, etc.)	12.3	1.5	9.1	10.9	9.6

Data provided in this table were extracted from five sources: Kim et al. (33, 35, 36), Jeon et al. (37), and Kang et al. (38). The units of the values in the table are in percentages (%).

Based on the responses to the question about adopting flexible working methods in the future, it appears that the preference for home-based telework or telecommuting is lower than that for other flexible working methods. Table 3 illustrates the preference for various flexible working methods to be added in the future and indicates that only 15.9% of respondents expressed a preference for home-based telework, whereas only 4.4% showed a preference for telecommuting. These data suggest that currently not only the usage of home-based telework and telecommuting is limited in Korea but these methods are also not highly favored as the preferred working methods for the future.

**Table 3:** Working methods to be added in the future (multiple responses).

Total (number of companies)	Home-based telework	Telecommuting	Selective working	Discretionary work system	Selective working hours	Staggered commuting
100.1 (15,986)	15.9 (2,536)	4.4 (696)	34.5 (5,520)	18.0 (2,882)	23.8 (3,801)	9.8 (1,573)

Jeon et al. (39).

The survey was conducted for the people in charge of personnel affairs working in the 5,049 companies selected through the stratified sampling method by business type and company size from companies with not less than five regular workers. The units of the numbers in the table are in percentages, and the units inside the parentheses are the number of companies.

#### 3.2.2 After the pandemic

After the COVID-19 pandemic hit Korea, there was a significant and rapid increase in the adoption of home-based telework, especially after the mass infection in Daegu. Heightened concerns about infections throughout the country led many companies to swiftly implement remote work arrangements. A report by a Korean company specializing in home-based telework and telecommuting solutions illustrates this trend. According to their findings, the usage of video conference services increased 15-fold within 1 month, from February 23, when the infection situation reached a critical level, to March 2 (40).

The implementation of a flexible working system, such as home-based telework, has been supported for SMEs in Korea through indirect labor cost subsidies. Since the outbreak of the COVID-19 pandemic, the number of businesses applying for this support has increased significantly, by approximately 20-fold. Among the various types of support applications, home-based telework constituted the majority, accounting for 52.5% (16,023 people), whereas telecommuting, which is a similar working method utilizing digital devices, accounted for only 1.3% (394 people) (41).

**Figure 6:**
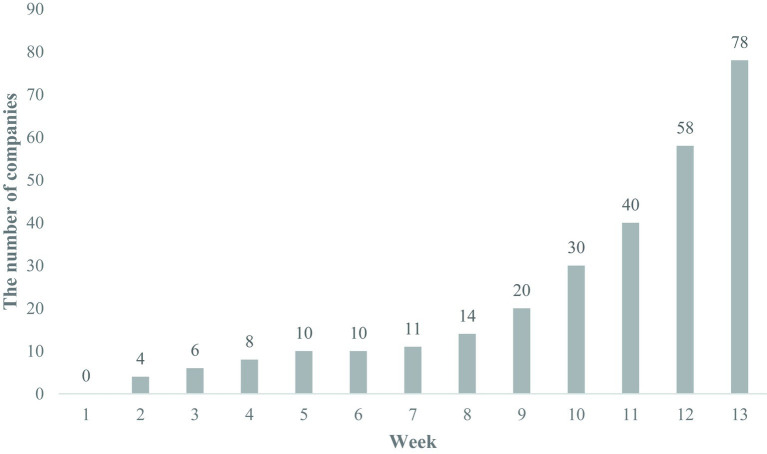
Status of the number of companies that have applied for home-based telework infrastructure construction costs (April 5, 2020; January 1–April 5, total). The Ministry of Employment and Labor (43).

As a response to the pandemic, the Korean government simplified support procedures for SMEs seeking to adopt flexible working methods. This involved expediting the evaluation of business applications, reducing the burden of evidence-based procedures, and expanding the pool of eligible workers (42). Consequently, the number of companies interested in implementing home-based telework has seen a rapid and substantial increase. Figure 6 shows the number of companies applying for telecommuting infrastructure.

#### 3.2.3 Nature of the change

As previously discussed, a significant difference exists between home-based telework in Korea and other advanced countries. While advanced countries have implemented and experimented with remote work to some extent, Korea has been more passive in adopting telework. Such countries have conducted trial and error during the implementation of home-based telework methods.

However, in response to the COVID-19 pandemic, there has been a rapid increase in the demand for IT services for home-based telework or telecommuting in Korea. This surge can be viewed as a temporary measure to cope with the pandemic, and home-based telework has been implemented without thoroughly testing its suitability for various job roles. For instance, employees in Korean call centers, who handle sensitive customer information and require immediate responses, were initially believed to be unsuitable for remote work. However, these jobs quickly transitioned to home-based telework when call centers became sites of mass infection (44). The pandemic has forced experimentation to determine whether home-based telework is suitable for Korea.

Some examples suggest that a surge in home-based telework may not be a temporary measure. Certain companies that have implemented telework for all employees have indicated their intentions to continue telecommuting. They envision a culture of business openness, sharing, and communication through the development of new business tools, possibly leading to their transformation into smart offices (45).

Considering this trend, it is important to explore whether home-based telework can become a method of business innovation not only during emergencies such as the COVID-19 pandemic but also in the era of the Fourth Industrial Revolution. The pandemic has acted as a turning point for organizations, prompting fundamental concerns over their methods of operation and job performance evaluation (46).

However, to determine whether the home-based telework method introduced abruptly due to the COVID-19 pandemic in Korea can last and expand in the post-pandemic era, various reviews and assessments should be conducted. We now consider the possibility of expanding home-based telework methods in Korea following this outbreak.

### 3.3 Review on the possibility of home-based telework methods to expand in Korea

#### 3.3.1 The possibility for change in the passive attitude of Korea

Conducting thorough reviews from various perspectives is crucial to determine the potential expansion of home-based telework methods in Korea following the pandemic. One key aspect is identifying jobs that can be effectively conducted through home-based telework. The survey examined the reasons for the previous lack of a flexible working system in Korea and found that over half of all jobs were deemed unsuitable for such a system. Table 4 provides insight into the reasons for not utilizing flexible working methods.

**Table 4:** Reasons why flexible working methods are not implemented.

Total (number of companies)	No suitable jobs	Difficulty in labor management such as worker evaluation	No workers who want it	Customer relation	Introduction procedure unknown	Introduction cost	Information security	Objection of employees
100 (565,465)	68.4 (386,719)	9.2 (51,787)	12.8 (72,278)	6.7 (38,166)	1.5 (8,586)	0.8 (4,560)	0.4 (2,297)	0.2 (1,071)

Jeon et al. (39).

The survey has been conducted for the people in charge of personnel affairs working in the 5,049 companies selected through the stratified sampling method by business type and company size from companies all over the country with not less than five regular workers. The units of the numbers in the table are in percentages, and the units inside the parentheses are the number of companies.

According to a previous survey, the main reasons for difficulties in encouraging flexible work included the nature of the job (52.3%) and organizational culture and environment (23.0%) (47). Collectively, these results indicate that the primary obstacles hindering the introduction of home-based telework are the lack of suitable job roles and the nature of the work itself.

#### 3.3.2 Judicial procedure required for the adoption of home-based telework in Korea

When introducing home-based telework in Korea, it is required to obtain the consent of individual workers or engage in consultation with worker groups before making any changes to working conditions. The process for implementing home-based telework includes the following scenarios:

First, if a provision concerning the implementation of home-based telework is already present in the collective agreement, employment rules,^1^ or labor contract, the company can implement home-based telework by issuing an order in accordance with the relevant provision. In such cases, individual workers are considered to have implicitly agreed to home-based telework, allowing the employer to implement it by exercising the right to personnel orders. Additionally, if the individual labor contract includes a provision allowing the employer to designate the workplace, the employer can introduce home-based telework by designating the employee’s home as the workplace without requiring any separate consent (Provision in Possession Method). However, such cases are rare in Korea, which has passively adopted home-based telework.

In situations without any provision related to implementing home-based telework, as previously mentioned, a company can only introduce home-based telework with the explicit consent of individual workers. As most Korean workplaces lack specific provisions for home-based telework, its implementation during the COVID-19 pandemic likely required obtaining the consent of individual workers. Nevertheless, if social distancing is imperative to prevent the spread of the infection, implementing home-based telework can be justified through consultation with workers or worker groups. Consequently, a stay-home order or home-based telework (transfer) order, in which the home becomes the workplace, can be reasonably issued^2^ (Right of Personnel Order Method).

Meanwhile, if home-based telework is officially introduced for all employees or a specific job group, working conditions, such as the service regulations related to home-based telework applied to relevant employees, must be modified. In such cases, conditions cannot be altered solely by the consent of individual workers; rather, home-based telework must be introduced by amending the employment rules that apply to all employees. However, if the proposed working conditions, such as the service regulations for home-based telework, are not more disadvantageous to workers than the existing working conditions, the opinion of the labor union shall be heard if such an organization represents the majority of the workers. In its absence, the collective opinion of the majority of workers should be considered (Article 94, Paragraph 1, Main Body of the Labor Standards Act, Normal Method to Change Employment Rules). However, if the proposed working conditions, such as service regulations for home-based telework, are more disadvantageous to workers than the existing working conditions (e.g., if the calculation of working hours is disadvantageous or wages decrease), the consent of either the head of the labor union representing the majority or, if there is no such labor union, that of a majority of the workers is required (Article 94, Paragraph 1, Proviso).

Notably, if the content of home-based telework being introduced is, in particular, more disadvantageous to workers, even if consent is obtained from the majority of the workers, the change in their labor contracts may not be effective if not all individual employees agree to it (Disadvantageous Change of Employment Rule Method). For example, if consent is obtained from a worker group regarding a decrease in commuting allowances and meal benefits but not all individual workers agree to the change, the introduction of home-based telework may be obstructed, and the pre-existing standards will continue to apply to those who have not agreed to it.^3^ Consequently, even after introducing home-based telework, the pre-existing working conditions continue to apply to workers who have not provided consent. Furthermore, if the group agreement stipulates a procedure for advance consultation or the consent of the labor union when introducing a new working method, such as home-based telework, that procedure must be followed accordingly.

### 3.4 Actual introduction of home-based telework and review of the possibility of post-pandemic expansion

As mentioned earlier, numerous articles have reported that home-based telework will expand in Korea because of the COVID-19 pandemic. However, introducing and expanding home-based telework in Korea may not be straightforward, because of the prevailing passive perception of remote work. The feasibility of continuing home-based telework after the outbreak can be assessed by examining the legal aspects related to its implementation in Korea, such as the process and specifics of the introduction.

Most home-based telework in Korea is likely to be established based on the employer’s right to issue personnel orders (the Right of Personnel Order Method), as seen in the judicial procedures investigated earlier. According to Korea’s Labor Standards Act, when employers exercise the right to issue personnel orders, such as granting leaves or transfers, there must be a justifiable reason without exception (Article 23, Paragraph 1 of the Labor Standards Act). Instructing employees to temporarily switch to home-based telework because of stringent social distancing measures to prevent epidemics is considered a justifiable use of the right to issue personnel orders. This was performed to protect the safety of the workplace and workers from infection. The necessity of preventing COVID-19 was acknowledged based on precedents set by the Supreme Court of Korea. Moreover, there were no disadvantages because this method was implemented without altering existing working conditions or salary levels. Furthermore, it was executed after undergoing the required consultation procedure under the principle of good faith, for instance, by conducting urgent consultations with workers. Thus, it can be deemed justifiable home-based telework.

There is no guarantee that most home-based telework mentioned earlier will continue after the outbreak. To assess their usefulness and efficiency, tests should be conducted during this period to identify potential productivity issues. Thus, an appropriate method for home-based telework could be developed and expanded after the outbreak. However, workers gradually returned to industrial sites after successfully preventing the spread of the pandemic (48). During this period of increased infection rates, home-based telework was implemented in Korea as a crisis management measure, but no research has been conducted to explore its potential as a long-term replacement for face-to-face work. This situation may have hindered the possibility of conducting such research. Moreover, home-based telework was not initially designed but rather implemented as an emergency response. While the number of companies applying for support for home-based telework infrastructure increased significantly during the pandemic, it is crucial to examine the nature of the changes in employment rules required for this support. If the changes only maintained the previous working conditions, they could be considered ordinary changes. However, if the conditions were lowered, this would be disadvantageous. The Korean government’s limitation on qualified applicants for home-based telework support for SMEs suggests that the possibility of working conditions being lowered for these businesses is low, indicating ordinary changes. Therefore, it is more likely that the expansion of home-based telework in Korea will be limited to SMEs that have applied for infrastructure support. Larger Korean companies that were highly competitive and productive before the pandemic may have remained passive in adopting home-based telework even after the outbreak.

The most significant concern in introducing home-based telework in Korea is the potential change in working conditions. The issue revolves around whether existing service regulations can be maintained when home-based teleworking is implemented uniformly for all employees or specific job groups. If teleworking is introduced without altering the previous working conditions, it is considered an ordinary change in employment rules, with the only difference being a change in the workplace. The procedure for this change typically involves considering the collective opinions of the worker group. However, if the change in working conditions negatively affects employees, for instance, if previous conditions are downwardly adjusted, the consent of the worker group and, in some cases, that of individual workers regarding changes in labor contracts may be required. Obtaining consent can be challenging in such cases. Various factors must be considered, such as whether home-based telework will be as productive as face-to-face work and whether certain benefits such as transportation allowances or meal subsidies should continue to be provided when the nature of home-based telework. For example, if commuting allowances and meal benefits are part of the regular salary paid to all employees in Korea,^4^ they are expected to be maintained even when transitioning to home-based telework. However, employers may hesitate to maintain these benefits if productivity is not guaranteed. Additionally, employers may be reluctant to pay salaries based on previous working hours because of the rigidities imposed by the Labor Standards Act, which is a method of payment per working hour, especially if there are no clear productivity or performance evaluation criteria.

Implementing blanket wage systems or discretionary work systems that allow for the flexible utilization of working hours and salaries would be ideal, but as discussed earlier, this is not a straightforward solution. Moreover, workers who have engaged in home-based telework may desire to return to face-to-face work if the home-based work environment changes or if they prefer working at the workplace. However, the procedures for transitioning between face-to-face and home-based telework cannot be resolved solely through legal principles related to disadvantageous changes in employment rules. Approaching an agreement between a company and an individual worker on the change in the working method as collective consent (protection) may lead to the possibility of future working method adjustments owing to the inflexibility of the system.

**Figure 7:**
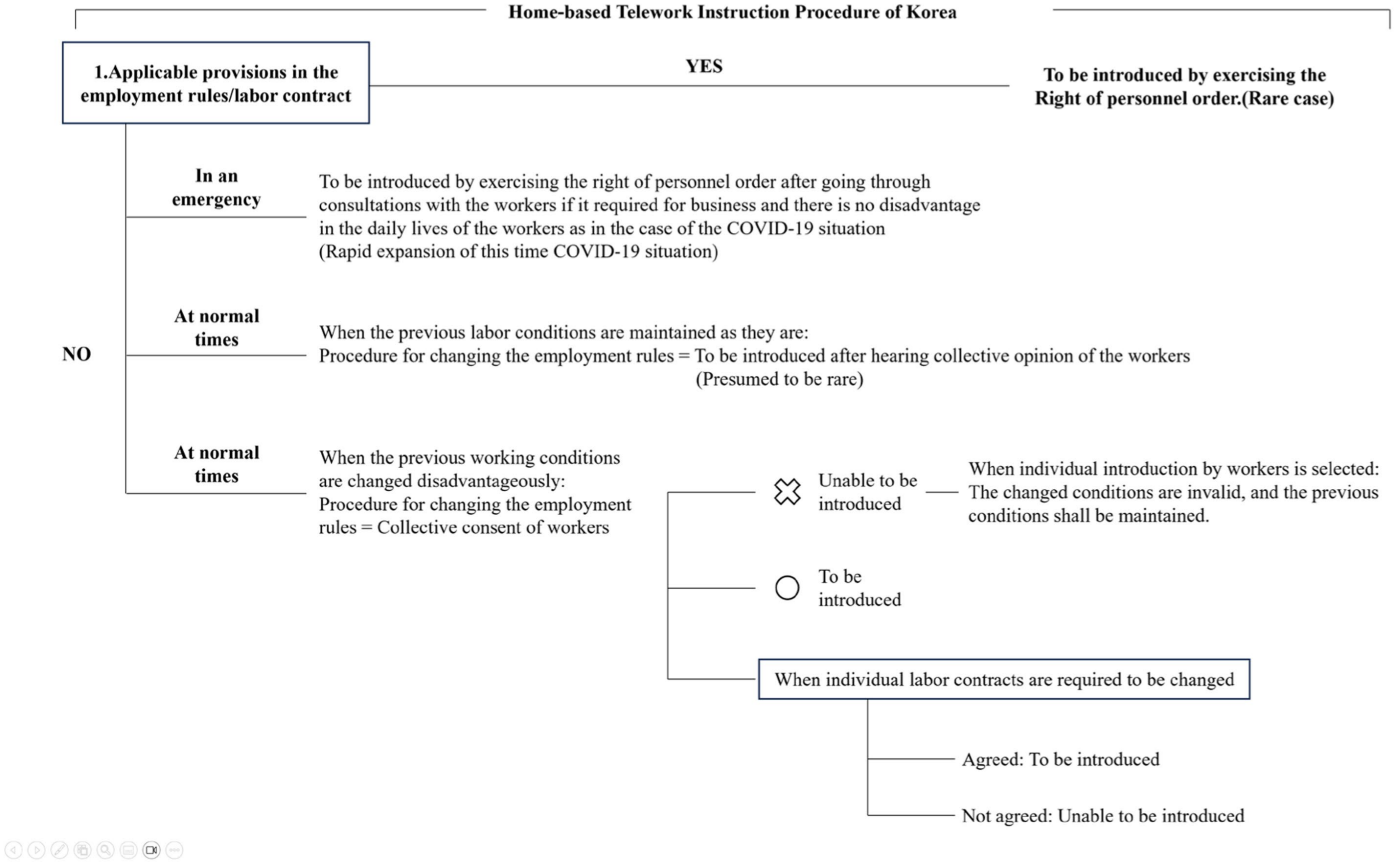
Home-based telework instruction procedure.

In a country such as Korea, with a pre-dominating face-to-face work culture, the introduction of home-based telework is likely to result in changes to existing working conditions. There might be concerns that teleworking may not guarantee the same level of productivity as face-to-face work because of potential issues such as poor productivity, the absence of a performance evaluation system, and difficulties in remote management, which are typically available in a physical workplace. Consequently, Korean employers may be more inclined toward adopting methods that involve certain disadvantageous changes for employees. One way to address this is through consultation between workers and management. However, worker-management relations in Korea often do not favor such cooperative approaches, leading many to prefer the resolution of such matters through legal means. Figure 7 illustrates the legal structure governing the adoption of home-based teleworking.

Indeed, during the COVID-19 pandemic, temporary home-based telework was implemented in Korea while maintaining previous working conditions. If such telework were accompanied by a reduction in salaries, many workers would likely choose to continue face-to-face work, even at the risk of infection, rather than accept the changes. Therefore, maintaining existing working conditions when introducing home-based telework in Korea is crucial for its successful adoption. A culture of negotiation and cooperation between workers and management will play a key role in navigating any potential downward changes in working conditions.

However, past worker-management relations in Korea have not been cooperative. There has been considerable distrust between workers and management, leading to emotional opposition over the division of benefits between employers and workers. According to the World Economic Forum’s Global Competitiveness Report 2019, Korea ranks 130th out of 141 countries in terms of “Cooperation in Labor-Employer Relations,” highlighting the challenges in achieving cooperation between the two parties (49). Given this situation, the confrontation between workers and management over the introduction of home-based telework in Korea can lead to difficulties in finding smooth resolutions.

The rapid increase in home-based telework in Korea since the COVID-19 pandemic has led to diverse assessments of its effectiveness. However, as most telework was implemented as a measure against infection, it primarily involved a change in the place of work without altering the existing working conditions, which helped avoid significant implementation issues. Additionally, as the Korean government managed to control the outbreak to some extent, many workers are returning to their workplaces despite the ongoing situation. Consequently, it is challenging to conclude that Korea has had the opportunity to fully embrace and implement home-based telework.

In Korea, a smooth and uniform introduction of home-based telework for all employees or specific workgroups can only be achieved if there are no changes to the existing working conditions. If any changes are made to working conditions that disadvantage workers, consent must be obtained following legal principles related to disadvantageous changes in employment rules. Even with the consent of the worker group, individual workers’ consent regarding changes in their respective labor contracts is also necessary. If home-based telework is introduced with the consent of individual workers without changing the employment rules, any changes that are not in line with existing working conditions will be invalidated, and workers should be treated under their previous conditions (as stated in Article 97 of the Labor Standards Act^5^).

**Figure 8:**
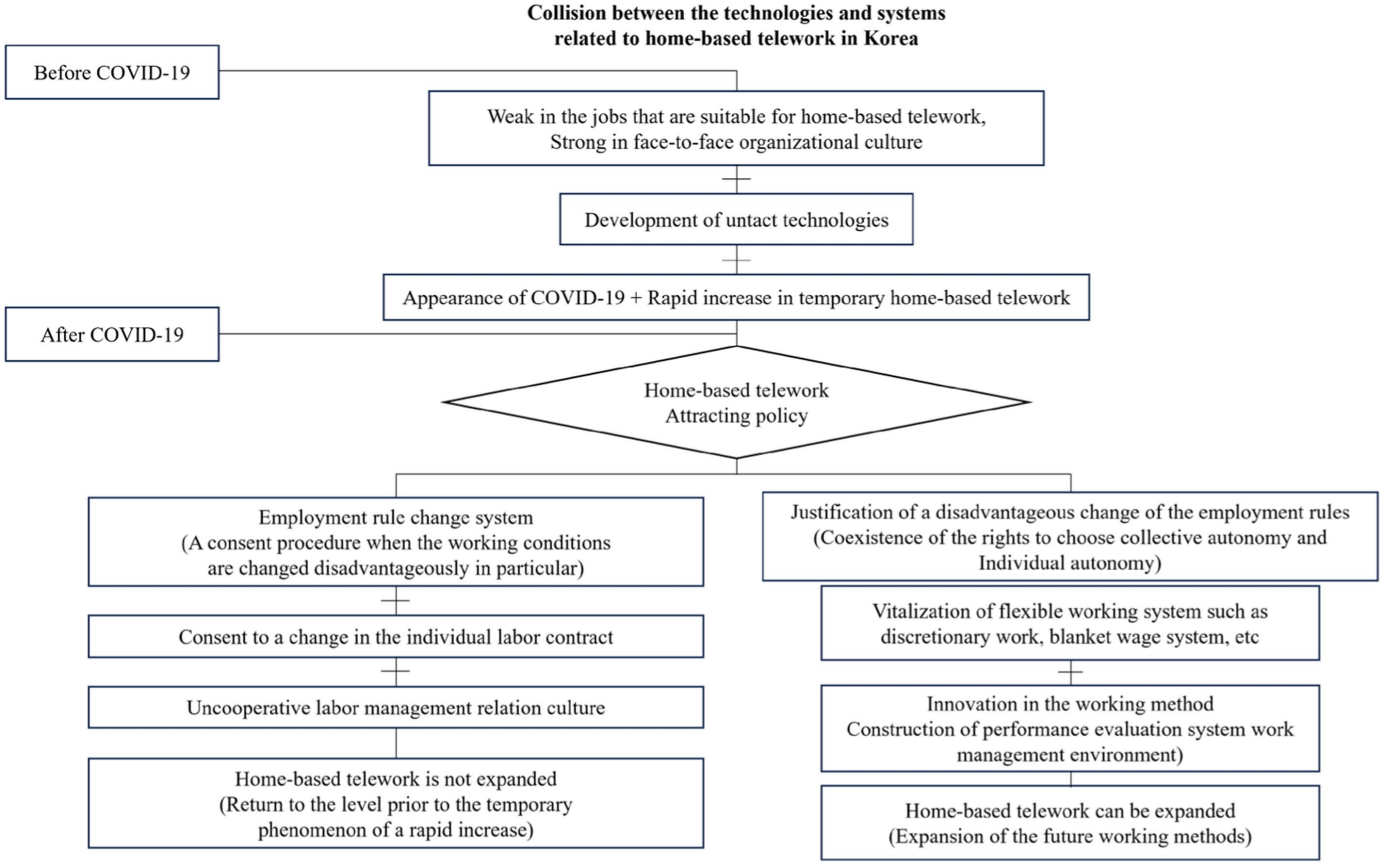
Collision between the technologies and systems related to home-based telework in Korea.

Although Korea possesses superior technical capabilities for developing “untact” technologies (technologies that enable contactless or remote interactions), the introduction and expansion of home-based telework require a cautious approach. To ensure successful implementation, it is essential to await suitable opportunities to conduct additional experiments and establish flexible operational conditions for the system to ensure a successful implementation. An environment for introducing improvements, such as telecommuting, should be prepared to facilitate collaboration with advanced technologies and ensure effectiveness. Figure 8 illustrates this process in detail.

## 4 Discussion

COVID-19 may become an endemic issue, particularly considering the recent discovery of asymptomatic infections (50). The stealth characteristics of infectious diseases, combined with the changes brought about by the Fourth Industrial Revolution, emphasize the need to transition to contactless methods, such as home-based telework. The COVID-19 situation has had various impacts on the labor market in Korea, sparking a preference for novel working methods over rigid face-to-face approaches. However, home-based telework is primarily a temporary measure to combat infections, and its introduction in Korea requires careful consideration and experimentation to assess its suitability.

A major obstacle to expanding home-based telework in Korea is the lack of institutional support mechanisms. The Korean legal principles concerning disadvantageous changes in employment rules must be adjusted to accommodate future working methods. The introduction of home-based telework should not be obstructed by worker groups’ failure to obtain consent for changes that may be perceived as disadvantageous. Instead, individual workers should be allowed to choose home-based telework based on their voluntary and genuine intentions. Protection should not hinder individual choices made freely. Additionally, if the consent of the worker group is obtained, pursuing further changes in individual labor contracts would be unnecessary, as coordinated worker and management autonomy has already been achieved. In the digital era, where jobs are evolving with greater autonomy and multidimensionality, group-based protection methods from traditional labor laws need to coexist with individual autonomy. A negotiation culture that ensures a win-win situation for both workers and management is crucial. Instead of strictly adhering to old worker protection methods, a more strategic and calculative negotiation culture is required to introduce home-based telework, which improves productivity and worker treatment.

The expansion of untact technology may foster the growth of home-based telework, but it may face challenges due to inflexible Korean labor laws, judicial precedents, and worker-management relations. If untact technology is well developed, but necessary legal and organizational changes do not occur, Korea may revert to the pre-pandemic period, or implementing change may take considerable time. Originally, home-based telework was more common in IT companies, such as Facebook and Twitter. However, the COVID-19 pandemic has forced the use of untact methods in all aspects of production, including the manufacturing industry. Companies leading the “new normal” are creatively redesigning productivity through home-based telework and innovative working methods. The rigidity of existing systems and organizational cultures that fail to keep up with technological advancements may become an additional hurdle for companies to overcome after a pandemic.

## 5 Conclusion

This study has critically examined the profound implications of the COVID-19 pandemic on the Korean labor market, specifically focusing on the dynamics of home-based telework. It is evident that home-based telework has emerged as a transformative response to the challenges posed by infectious diseases, marked by advantages such as increased productivity and an enhanced safety framework. However, the long-term viability of home-based telework after the pandemic remains uncertain. To become a stable and enduring system in the long run, it requires legal and social consensus on issues such as labor-management agreements, legal procedures, and job conditions. Moreover, there is a need for the existing institutional framework to evolve toward a more forward-looking direction. As technology, such as untact technology, advances, the labor market needs to adapt accordingly. Lack of innovative changes in the legal and social aspects could hinder the technology’s ability to establish a suitable work environment.

In this light, this study provides comprehensive insights into the contemporary work landscape post-pandemic and offers strategic guidance for optimizing the potential of home-based telework while adeptly addressing its multifaceted challenges. It sets the stage for a more efficient, harmonious, and productive workforce in the era beyond the COVID-19 pandemic. The implications of this research are instrumental for organizations and policymakers seeking to navigate the evolving work dynamics and enhance the well-being of both employees and employers.

This study focuses on the early changes during the onset of the COVID-19 pandemic and perspectives regarding those changes. The global spread of the coronavirus has led to the activation of a culture emphasizing non-contact practices. A prominent transformation in work patterns, highlighting the significance of non-contact interactions, is the adoption of telecommuting or remote work. However, it remains uncertain whether the utilization of such work forms will continue to increase after the conclusion of the COVID-19 pandemic. Therefore, future research may need to investigate changes in work patterns over an extended period. Additionally, considering the evolving nature of industry structures and work patterns over time, research taking into account both job characteristics and industry characteristics is deemed necessary.

## Data availability statement

Publicly available datasets were analyzed in this study. This data can be found at: https://www.kwdi.re.kr/, https://www.moel.go.kr/english/, http://www.fki.or.kr/Common/Download.aspx?id=2dfd975c-5e1f-48aa-a550-595211ae3d8f.

## Author contributions

JC: Conceptualization, Writing – original draft, Writing – review & editing. SL: Conceptualization, Writing – original draft, Writing – review & editing. BP: Data curation, Writing – original draft, Writing – review & editing.

## Footnotes

^1^Employment rules specify the working conditions to be applied uniformly to the workers in a business place regardless of the designation. In Korea, the business places that employ more than 10 workers are obliged to establish employment rules (Article 94, Main Body of the Labor Standards Act). Although employment rules contain the working conditions and working discipline of the workers in business places existing in Korea and Japan, they also can also nullify the relevant contract provisions if the labor contract concluded by an individual worker falls short of the standard set by the employment rules (Article 97 of the Labor Standards Act).

^2^Article 23, Paragraph 1, of the Labor Standards Act stipulates, “The employer shall not dismiss…leave of absence, or transfer…without good cause.” Whether a personnel change, such as an order to work from home or a stay-home order (transfer or leave of absence), is based on a good cause is determined through business necessity, disadvantages in life, and the consultation procedure required under the principle of good faith (Supreme Court, April 11, 2000, Pronouncement of Ruling No. 99du2963; Supreme Court, Sep. 10, 2009, Pronouncement No. 2009du10440, etc.)

^3^Supreme Court, November 14, 2019, Pronouncement of Ruling No. 2018da200709 is a ruling that states that the application of the salary peak system to the relevant worker is not effective if the employer fails to obtain the consent of the individual worker who is against the introduction of such a system on the change in the labor contract, even though the labor union organized with a majority of the workers has agreed on such an introduction, which decreases the salary after a certain age. Recently, there has been considerable controversy regarding this ruling, and it has been argued, for example, that it is a decision that ignores the uniform and across-the-board nature, an important function of employment rules. For example, Hyungbae Kim, Disadvantageous Change of Employment Rules and Advantageous Provisions of Labor Contract, Society of Labor Law Theory and Profession, Labor Law Forum No. 29, 2020. pp. 4 onward. Joonhee Lee, Superiority and Inferiority Relation between the Effects of Employment Rules and Labor Contract – Around the Supreme Court Pronouncement of Ruling No. 2018da200709 dated November 14, 2019, Society of Labor Law Theory and Profession, Collection of Treatises related to Labor Law No. 48, 2020.4. pp. 383 onward.

^4^Supreme Court 1996.5.10. Pronouncement No. 95da2227, etc.

^5^Article 97 of the Labor Standards Act: If the working conditions set by a labor contract fall short of the working conditions prescribed in the employment rules, the part of the labor contract shall be invalid. In this case, for the part invalidated, the standard prescribed in the employment rules shall be complied with.

## References

[1] Pérez MP Sánchez AM De Luis Carnicer MP. The organizational implications of human resources managers’ perception of teleworking. Pers Rev. (2003) 32:733–55. doi: 10.1108/00483480310498693

[2] Bailey D Kurland NB. A review of telework research: findings, new directions, and lessons for the study of modern work. J Organ Behav. (2002) 23:383–400. doi: 10.1002/job.144

[3] Fonner KL Roloff ME. Why teleworkers are more satisfied with their jobs than are office-based workers: when less contact is beneficial. J Appl Commun Res. (2010) 38:336–61. doi: 10.1080/00909882.2010.513998

[4] Sánchez AM Pérez-Pérez M Vela-Jiménez MJ de-Luis-Carnicer P. Telework adoption, change management, and firm performance. J Organ Chang Manag. (2008) 21:7–31. doi: 10.1108/09534810810847011

[5] Ammons SK Markham WT. Working at home: experiences of skilled WHITE COLLAR workers. Sociol Spectr. (2004) 24:191–238. doi: 10.1080/715726145

[6] Bjursell C Bergmo-Prvulovic I Hedegaard J. Telework and lifelong learning. Front Sociol. (2021) 6:642277. doi: 10.3389/fsoc.2021.642277, PMID: 33869587 PMC8022557

[7] Cooper CD Kurland NB. Telecommuting, professional isolation, and employee development in public and private organizations. J Organ Behav. (2002) 23:511–32. doi: 10.1002/job.145

[8] Golden TD Veiga JF. The impact of superior–subordinate relationships on the commitment, job satisfaction, and performance of virtual workers. Leadership Q. (2008) 19:77–88. doi: 10.1016/j.leaqua.2007.12.009

[9] Lapierre L Van Steenbergen EF Peeters MCW Kluwer ES. Juggling work and family responsibilities when involuntarily working more from home: a multiwave study of financial sales professionals. J Organ Behav. (2015) 37:804–22. doi: 10.1002/job.2075

[10] Madsen SR. The effects of home-based teleworking on work-family conflict. Hum Resour Dev Q. (2003) 14:35–58. doi: 10.1002/hrdq.1049

[11] Wilson M Greenhill A. Gender and teleworking identities in the risk society: a research agenda. N Technol Work Employ. (2004) 19:207–21. doi: 10.1111/j.1468-005x.2004.00138.x

[12] Eddleston KA Mulki JP. Toward understanding remote workers’ management of work–family boundaries: the complexity of workplace embeddedness. Group Org Manag. (2015) 42:346–87. doi: 10.1177/1059601115619548

[13] Dutcher EG. The effects of telecommuting on productivity: an experimental examination. The role of dull and creative tasks. J Econ Behav Organ. (2012) 84:355–63. doi: 10.1016/j.jebo.2012.04.009

[14] Boell SK Ćećez-Kecmanović D Campbell J. Telework paradoxes and practices: the importance of the nature of work. N Technol Work Employ. (2016) 31:114–31. doi: 10.1111/ntwe.12063

[15] Kaluza AJ Van Dick R. Telework at times of a pandemic: the role of voluntariness in the perception of disadvantages of telework. Curr Psychol. (2022) 42:18578–89. doi: 10.1007/s12144-022-03047-5, PMID: 35382038 PMC8970638

[16] Alipour J Fadinger H Schymik J. My home is my castle – the benefits of working from home during a pandemic crisis. J Public Econ. (2021) 196:104373. doi: 10.1016/j.jpubeco.2021.104373

[17] Belzunegui-Eraso A Erro-Garcés A. Teleworking in the context of the COVID-19 crisis. Sustainability. (2020) 12:3662. doi: 10.3390/su12093662

[18] Kniffin KM Narayanan J Anseel F Antonakis J Ashford SP Bakker AB. COVID-19 and the workplace: implications, issues, and insights for future research and action. Am Psychol. (2021) 76:63–77. doi: 10.1037/amp0000716, PMID: 32772537

[19] Okubo T Inoue A Sekijima K. Teleworker performance in the COVID-19 era in Japan. Asian Econ Papers. (2021) 20:175–92. doi: 10.1162/asep_a_00807

[20] Haider M Anwar A. The prevalence of telework under Covid-19 in Canada. Inf Technol People. (2022) 36:196–223. doi: 10.1108/itp-08-2021-0585

[21] Nguyen MH. Factors influencing home-based telework in Hanoi (Vietnam) during and after the COVID-19 era. Transportation. (2021) 48:3207–38. doi: 10.1007/s11116-021-10169-5, PMID: 33518829 PMC7821989

[22] Okubo T. Telework in the spread of COVID-19. Inf Econ Policy. (2022) 60:100987. doi: 10.1016/j.infoecopol.2022.100987

[23] Carillo K Cachat-Rosset G Marsan J Saba T Klarsfeld A. Adjusting to epidemic-induced telework: empirical insights from teleworkers in France. Eur J Inf Syst. (2020) 30:69–88. doi: 10.1080/0960085x.2020.1829512

[24] Moens E Lippens L Sterkens P Weytjens J Baert S. The COVID-19 crisis and telework: a research survey on experiences, expectations and hopes. Eu J Health Econ. (2021) 23:729–53. doi: 10.1007/s10198-021-01392-z, PMID: 34761337 PMC8580807

[25] Vyas L. “New normal” at work in a post-COVID world: work–life balance and labor markets. Polic Soc. (2022) 41:155–67. doi: 10.1093/polsoc/puab011

[26] Lang G Hofer-Fischanger K. Factors associated with the implementation of health-promoting telework from the perspective of company decision makers after the first COVID-19 lockdown. J Public Health. (2022) 30:2373–87. doi: 10.1007/s10389-022-01717-z, PMID: 35530416 PMC9064540

[27] Dingel JI Neiman B. How many jobs can be done at home? J Public Econ. (2020) 189:104235. doi: 10.1016/j.jpubeco.2020.104235, PMID: 32834177 PMC7346841

[28] Bursztynsky Jessica. “AT&T asks all employees to work from home if they can due to coronavirus.” CNBC (2020). Available at:https://www.google.com/amp/s/www.cnb c.com/amp/2020/03/13/coronavirus-att-asks-all-employees-to-work-from-home-if-they-can.html (Accessed May 21, 2020).

[29] Palmer Annie. "Amazon asks all employees to work from home." CNBC (2020). Available at:https://www.cnbc.com/2020/03/12/amazon-tells-all-employees-to-stay-home-amid-coronavirus-fears.html (Accessed May 21, 2020).

[30] Kim Daewoo. Application for flexible working system is rapidly increasing as home-based telework increases in response to COVID-19. Herald Economy, March 6 (2020). Available at:http://biz.heraldcorp.com/view.php?ud=20200306000054 (Accessed May 21, 2020).

[31] Park Sungjin. College students will take on-line lessons at home in principle until COVID-19 ends. Yonhap News (2020). Available at:https://www.yna.co.kr/view/AKR20200302152700004 (Accessed May 21, 2020).

[32] Korea Chamber of Commerce. (2016). Survey on introduction of a flexible working system by companies. Available at:http://www.korcham.net/nCham/Service/Economy/appl/KcciReportDetail.asp?SEQ_NO_C010=20120930850&CHAM_CD=B001 (Accessed May 21, 2020).

[33] YoungOck K Jongsoog K Seonhaeng L. Survey on compatibility conditions of work and family life. The Ministry of Employment and Labor. (2015)

[34] Messenger J Llave OV Gschwind L Boehmer S Vermeylen G Wilkens M. (2017). Working anytime, anywhere: the effects on the world of work. Joint ILO–Eurofound report:17. Available at:https://www.eurofound.europa.eu/publications/report/2017/working-anytime-anywhere-the-effects-on-the-world-of-work (Accessed May 21, 2020).

[35] YoungOck K Seunghyun L Seonhaeng L. Survey on compatibility conditions of work and family life. The Ministry of Employment and Labor. (2016)

[36] YoungOck K Jongsoog K Seonhaeng L. Survey on compatibility conditions of work and family life. The Ministry of Employment and Labor. (2017)

[37] Minjung K Jongsoog K Nanjue K Seonhaeng L Soyong K. Survey on compatibility conditions of work and family life. The Ministry of Employment and Labor. (2018)

[38] Kitaek J Jongsoog K Minjung K Nanjue K Seonhaeng L Soyong K. Survey on compatibility conditions of work and family life. The Ministry of Employment and Labor. (2019)

[39] Jeon K. 2017 survey on compatibility conditions of work and family life. The Ministry of Employment and Labor (Korea Women’s Development Institute). (2019)

[40] Kim Sungsoo. “Rapid increase in the demand for the home-based telework related solutions due to expansion of COVID-19 infection,” IT Daily, March 26 (2020). Available at:https://www.itdaily.kr/news/articleView.html?idxno=100175 (Accessed May 21, 2020).

[41] The Ministry of Employment and Labor of South Korea. “The application for the flexible working system has increased greatly in response to COVID-19.” The Ministry of Employment and Labor News, Official Blog of the Ministry of Employment and Labor (2020). Available at:https://blog.naver.com/molab_suda/221840527708 (Accessed May 21, 2020).

[42] The Ministry of Employment and Labor of South Korea. “The application procedure for the flexible working system will be simplified for prevention of COVID-19.” Press Release, Web Site of the Ministry of Employment and Labor (2020). http://www.moel.go.kr/news/enews/report/enewsView.do?news_seq=10756 (Accessed May 21, 2020).

[43] The Ministry of Employment and Labor of South Korea, “Firms for excellent home-based telework.” Press release, Web Site of the Ministry of Employment and Labor, (2020). Available at:http://www.moel.go.kr/news/enews/report/enewsView.do?news_seq=10881 (Accessed May 21, 2020).

[44] Kim Keechan. “Call centers, also home-based telework···COVID-19 has broken ‘9 to 6 working hours’ which the government has failed to break,” Joongang Ilbo, March 16 (2020). Available at:https://news.joins.com/article/23731139 (Accessed May 21, 2020).

[45] Ha Sungyoung. (2020). Work from home even after the COVID-19 situation ends, COVID-19 reversal of companies, why?. Joongang Ilbo. Available at:https://news.joins.com/article/23719340 (Accessed May 21, 2020).

[46] Myeong Soonyoung. “Korea is working from home,” Maeil Business Newspaper (2020). Available at:https://www.mk.co.kr/news/economy/view/2020/03/269268/ (Accessed May 21, 2020).

[47] Yoon K. Study on the ways to improve White Collar working system in the smart work period. Good Workplace Research Institute, the Korea Economic Daily, Research Report. (2014):26.

[48] Kwak Doyoung. “‘Back to the company again’… IT industry, home-based telework is released one after another,” Dong-A Newspaper (2020). Available at:http://www.donga.com/news/article/all/20200406/100529605/1 (Accessed May 21, 2020).

[49] Schwab Klaus. (2019). “The Global Competitiveness Report 2019.” World Economic Forum:324. Available at:http://www3.weforum.org/docs/WEF_TheGlobalCompetitivenessReport2019.pdf (Accessed May 21, 2020).

[50] Begley Sharon. “Experts envision two scenarios if the new coronavirus isn’t contained.” STAT (2020). Available at:https://www.statnews.com/2020/02/04/two-scenarios-if-new-coronavirus-isnt-contained/ (Accessed May 21, 2020).

